# Segregation and Heritability of Male Sterility in Populations Derived from Progeny of Satsuma Mandarin

**DOI:** 10.1371/journal.pone.0162408

**Published:** 2016-09-02

**Authors:** Shingo Goto, Terutaka Yoshioka, Satoshi Ohta, Masayuki Kita, Hiroko Hamada, Tokurou Shimizu

**Affiliations:** Division of Citrus Research, Institute of Fruit Tree and Tea Science, NARO, Shizuoka, Japan; USDA-ARS Southern Regional Research Center, UNITED STATES

## Abstract

Male sterility derived from Satsuma mandarin (*Citrus unshiu*) has been used in Japanese citrus breeding programs to obtain seedless cultivars, which is a desirable trait for consumers. Male sterility has often been evaluated by anther development or pollen fertility; however, the inheritance and heritability of male sterility derived from Satsuma is poorly understood. In this study, we investigated the mode of inheritance and broad-sense heritability of male sterility derived from Satsuma. Initially, we evaluated the total number of pollen grains per anther and apparent pollen fertility, as indicated by lactophenol blue staining, in 15 citrus cultivars and selections to understand the male sterility of Satsuma. The results indicated that male sterility was primarily caused by decreased number of pollen grains per anther in progeny of Satsuma. We also evaluated these traits in three F_1_ populations (hyuganatsu × ‘Okitsu No. 56’, ‘Okitsu No. 46’ × ‘Okitsu No. 56’ and ‘Okitsu No. 46’ × ‘Kara’), of which the parents are derived from Satsuma. Individuals in these populations showed strong segregation for number of pollen grains per anther. The apparent fertility of pollen also showed segregation but was almost constant at 70%–90%. The estimated broad-sense heritability for the number of pollen grains per anther was as high as 0.898 in the ‘Okitsu No. 46’ × ‘Okitsu No. 56’ and ‘Okitsu No. 46’ × ‘Kara’ populations. These results indicated that the number of pollen grains per anther primarily determined male sterility among progeny of Satsuma, and this trait was inherited by the progeny. Development of DNA markers closely linked to male sterility using the F_1_ populations of ‘Okitsu No. 46’ × ‘Okitsu No. 56’ and ‘Okitsu No. 46’ × ‘Kara’ is expected to contribute to the breeding of novel seedless citrus cultivars.

## Introduction

Male sterility in plants can be defined as failure of pollen grain development or function. Male sterility is categorized into genic, cytoplasmic or genetic-cytoplasmic types [1]. Genic male sterility is caused by a nuclear gene alone. Cytoplasmic male sterility is caused by a mitochondrial male sterility gene and is inherited from the seed parent. Cytoplasmic male sterility may be canceled by nuclear fertility-restoration genes, thereby fertility is recovered. Genetic-cytoplasmic male sterility is caused by cooperative action of cytoplasmic sterility and nuclear male-sterility genes [1]. Among these sterility types, cytoplasmic male sterility has been used for F_1_ hybrid breeding in self-pollinating crop species and vegetables. In addition, genes associated with cytoplasmic male sterility and its restoration have been identified in a variety of crops [2].

Citrus are among the most economically important fruit trees and are widely cultivated in temperate and subtropical regions. Seedlessness is a desirable trait for fresh and processed fruit in the citrus industry. Recent studies highlight the importance of male sterility in combination with female sterility and parthenocarpy to obtain seedless [3,4]. Satsuma, in which high frequency and stability of seedlessness is achieved by a combination of male sterility, female sterility, and parthenocarpy [4,5], has often been used in Japanese citrus breeding programs and several seedless cultivars have been selected [6]. Accordingly, male sterility is considered an important cause of seedlessness in progeny of Satsuma [3,4].

Satsuma (*Citrus unshiu*) and its progeny, such as ‘Kiyomi’, ‘Okitsu No. 46’ or ‘Harehime’, show severe male sterility characterized by undeveloped anthers [5,7–9]. Therefore, male sterility among progeny of Satsuma has been evaluated by focusing on anther development [9,10]. It has been suggested that progeny of Satsuma show genetic-cytoplasmic male sterility caused by the cooperative action of both cytoplasmic and nuclear genes derived from Satsuma [10,11]. These studies led following studies that the presumed male sterility of Satsuma would be achieved by CMS. Guo and colleagues had reported efforts to introduce the male sterility of Satsuma to other varieties by transferring cytoplasm of it by cybrid formation [12–15]. Though these studies did not confirm male sterility in these cybrid plants, recent studies by Zhen et al reported severe retardation of anther was observed in the cybrid pomelo, and proposed interaction of dysfunctional mitochondrial genes to suppress nuclear genes to suppress anther formation [16].

In the original report by Yamamoto [10], they proposed a possible role of cytoplasm for the male sterility, but they did not specify the role of nuclear gene. In the previous report of them, however, Yamamoto et al hypothesized an involvement of two nuclear genes for the male sterility of several citrus varieties [11]. Furthermore, Nakano et al reported that the anther phenotype is controlled by more than three nuclear genes in Satsuma [9]. These observations suggest that male sterility of Satsuma is inherited by progeny. Cybrid formation and regeneration from cell fusion requires sophisticated technique to obtain a plant transferred cytoplasm from Satsuma. Introducing the male sterility of Satsuma by usual cross hybridization technique is an attractive alternative approach when the male sterility was regulated by nuclear gene. Developing a DNA marker linked to such male sterility will facilitate seedless breeding of citrus.

Nakano et al. evaluated male sterility by focusing on the development of anthers and categorized the phenotype to only three ranks [9]. However, it was not evident whether total pollen grain number or pollen fertility primarily determines the male sterility of Satsuma and progeny derived from Satsuma. In addition, it is unclear whether total pollen grain number and pollen fertility are qualitative or quantitative traits. Determining the mode of inheritance of this type of male sterility, and estimating its heritability are highly anticipated to promote seedless breeding in citrus. Several cultivars and selections that show male sterility were selected from among progeny derived from Satsuma at the Institute of Fruit Tree and Tea Science, NARO [6]. These cultivars and selections represents useful materials for the study of citrus male sterility.

It is generally accepted that a quantitative trait is governed by a combination of genetic and environmental effects. The variance of a phenotype in a cross population includes the variance caused by genetic and environmental effects. The proportion of the phenotypic variance caused by all potential sources of genetic variance indicates broad-sense heritability. That is, broad-sense heritability represents an index of the proportion of phenotypic variance explainable by genetic effects [17]. Therefore, estimation of heritability facilitates selection of promising crosses and improves the efficiency of a breeding program. Estimating heritability also enables development of DNA markers linked to a trait of interest for marker-assisted selection. A trait that shows high heritability will make an important contribution to citrus breeding; therefore, accurate and reliable estimation of genetic effects on phenotypic variance is essential.

Previously, we established a method to evaluate male sterility in detail by focusing on the number of pollen grains per anther and the apparent grain fertility in citrus. We also evaluated male sterility in three F_1_ populations (hyuganatsu × ‘Okitsu No. 56’, ‘Okitsu No. 46’ × ‘Okitsu No. 56’ and ‘Okitsu No. 46’ × ‘Kara’) that were progeny of male sterile ‘Okitsu No. 46’ and ‘Okitsu No. 56’, but the data were obtained in a preliminary trial in only 1 year of evaluation [18]. In the present study, we investigated the male sterility of Satsuma by focusing on the number of pollen grains per anther and the apparent grain fertility to clarify the mode of inheritance of male sterility. We studied three F_1_ populations (hyuganatsu × ‘Okitsu No. 56’, ‘Okitsu No. 46’ × ‘Okitsu No. 56’ and ‘Okitsu No. 46’ × ‘Kara’) using data obtained over 2 years of evaluation, and estimated the broad-sense heritability of the traits. We demonstrated that the number of pollen grains per anther primarily determines male sterility in progeny of Satsuma, and this trait is inherited by the progeny of ‘Okitsu No. 46’.

## Materials and Methods

### Plant materials

Citrus cultivars and selections used in this study were part of germplasm collections or breeding populations maintained by the Division of Citrus Research, Institute of Fruit Tree and Tea Science, NARO (Table 1). ‘Okitsu Wase’ is a selection of Satsuma derived from a nucellar embryo [19]. Hassaku and hyuganatsu are chance seedlings selected in Hiroshima prefecture and Miyazaki prefecture in Japan, respectively. ‘Trovita’ is a selection of sweet orange. ‘Kiyomi’, ‘Harehime’, ‘Sweet Spring’, ‘Tamami’, ‘Shiranuhi’ ‘Mihaya’, ‘Okitsu No. 46’ and ‘Okitsu No. 56’ are derived from Satsuma selected at the Institute of Fruit Tree and Tea Science, NARO. ‘Kara’ is a hybrid of Satsuma selected by Dr H. B. Frost of the University of California Citrus Research Center, Riverside, CA, USA. Three F_1_ populations (hyuganatsu × ‘Okitsu No. 56’, ‘Okitsu No. 46’ × ‘Okitsu No. 56’ and ‘Okitsu No. 46’ × ‘Kara’) were provided for this study (Table 2). The seedlings of these populations were grafted onto trifoliate orange rootstock as a single replicate in April 2012 at the orchard of Okitsu, Shizuoka (35°3′ N, 138°31′ E; annual rainfall, 2368 mm; 8 m asl; annual average temperature, 16.3°C).

**Table 1:** Plant materials used in the study.

Cultivar/selection	Scientific name or hybrid parentage	Accession no.
Satsuma ‘Okitsu Wase’	*Citrus unshiu* Marc.	170630
Hyuganatsu	*C*. *tamurana* hort. ex Tanaka	117317
Hassaku	*C*. *hassaku* hort. ex Tanaka	117286
Sweet orange ‘Trovita’	*C*. *sinensis* (L.) Osbeck	172154
Rough lemon	*C*. *jambhiri* Lush	n.a.
‘Meyer lemon’	*C*. *meyeri* hort. ex Tanaka	n.a.
‘Kiyomi’	Hybrid (Satsuma ‘Miyagawa Wase’ × sweet orange ‘Trovita’)	115521
‘Okitsu No. 46’	Hybrid (‘Sweet Spring’ × sweet orange ‘Trovita’)	n.a.
‘Harehime’	Hybrid (‘E-647’* × Satsuma)	n.a.
‘Sweet Spring’	Hybrid (Satsuma × hassaku)	168866
‘Tamami’	Hybrid (‘Kiyomi’ × ‘Wilking’)	n.a.
‘Okitsu No. 56’	Hybrid (‘Okitsu No. 45’* × ‘Nou No. 5’*)	n.a.
‘Shiranuhi’	Hybrid (‘Kiyomi’ × ponkan#)	117159
‘Mihaya’	Hybrid (‘Tsunonozomi’ × ‘No. 1408’*)	n.a.
‘Kara’	Hybrid (Satsuma ‘Owari’ × King mandarin#)	113158

The Accession No. column presents the accession ID (JP number) in the NIAS Genebank. “n.a” indicates that an accession number has not been registered in the NIAS Genebank.

*: ‘E-647’ (‘Kiyomi’ × ‘Osceola’), ‘Okitsu No. 45’ (‘Kiyomi’ × ‘Wilking’), ‘Nou No. 5’ (‘Lee’ × mukaku kishu#), ‘No. 1408’ ((‘Encore’ × Satsuma ‘Okitsu Wase’) × (‘Kiyomi’ × Iyo#)).

^#^: ponkan (*Citrus reticulata* Blanco, 113178), King mandarin (*Citrus nobilis* Lour.), mukaku kishu (*Citrus kinokuni* hort. ex Tanaka), iyo (*Citrus iyo* hort. ex Tanaka).

**Table 2:** F_1_ populations used in the study.

Cross combination	Grafting year	Number of seedlings	Number of seedlings that flowered	
			2014	2015
Hyuganatsu × ‘Okitsu No. 56’	2012	60	43	49
‘Okitsu No. 46’ × ‘Okitsu No. 56’	2012	57	34	49
‘Okitsu No. 46’ × ‘Kara’	2012	36	22	34

First flowering was observed in 2014 after grafting of all individuals in each population.

### Evaluation of male sterility

To evaluate male sterility, we focused on the total number of pollen grains per anther and the pollen grain fertility. Three typical flowers per individual seedling were collected immediately after full bloom during anthesis in May 2014 and 2015. The flowers were stored in plastic containers with silica gel at −30°C until evaluation. The anthers were removed from each flower with forceps, and then immersed into 50 μl of lactophenol blue solution (Merck Millipore, Billerica, MA, USA) with shaking at 300 rpm for 1 h at room temperature. 10–15 anthers were used in an individual flower which has less than 500 of pollen grains per anther, and 3–5 anthers were used in that has more than 500 of pollen grains per anther. The anthers were dehisced with forceps, then shaken at the same speed for an additional two hours. An aliquot of the pollen grains dispersed in lactophenol blue solution was put on a Thoma hemocytometer, and then digital images were taken under a microscope (Eclipse 80iR, NIKON, Japan). The number of observed pollen grains was counted from the digital images using ImageJ software (http://imagej.nih.gov/ij/) with a plug-in “cell counter”. The number of pollen grains per anther was converted from the average number of grains in digital images and determined from three flowers in an individual seedling. The ratio of densely stained pollen grains to the total number of pollen grains (the sum of stained and unstained pollen grains) was regarded as the apparent pollen fertility in this study. The pollen grains were counted up to 30–50 in an individual flower which has less than 500 of pollen grains per anther, and 50–200 in that has more than 500 of pollen grains per anther for calculating the apparent pollen fertility. The apparent fertility were determined from three flowers in an individual seedling (the detail of this procedure was reported in [18]).

To measure anther length, all anthers from each flower were placed under a SZ61 stereomicroscope (Olympus, Tokyo, Japan) equipped with a digital camera. The anther length was measured from digital images using ImageJ software (http://imagej.nih.gov/ij/).

### Estimation of broad-sense heritability

Broad-sense heritability of the number of pollen grains per anther and apparent pollen fertility were estimated following the method of Yamada et al [20,21]. The observed number of pollen grains per anther was square-root transformed, and apparent pollen fertility was arcsine transformed. The normal distribution of the transformed data was confirmed with a one-sample Kolmogorov–Smirnov test [22]. The transformed data were subjected to analysis of variance (ANOVA) using the ‘stats’ package version 3.4.0 (https://stat.ethz.ch/R-manual/R-devel/library/stats/html/00Index.html) for R with the following model:

$$P_{ij}=\mu +G_{i}+Y_{j}+R_{ij}$$

where *P*_*ij*_ = the phenotypic value of the *i*th genotype in the *j*th year, μ = the overall mean, *G*_*i*_ = the effect of the *i*th genotype, *Y*_*j*_ = the effect of the *j*th year, and *R*_*ij*_ = the residual effect in the *i*th genotype of the *j*th year. The result of the ANOVA provided the genetic (σ_*g*_^2^), yearly (σ_*y*_^2^) and residual (σ_*r*_^2^) variances. The environmental variance (σ_*e*_^2^) for *n* years was estimated as σ_*e*_^2^ = (σ_*y*_^2^ + σ_*r*_^2^)/*n*. The broad-sense heritability (*h*_*B*_^2^) was calculated as *h*_*B*_^2^ = σ_*g*_^2^/(σ_*g*_^2^ + σ_*e*_^2^).

## Results and Discussion

### Evaluation of male sterility in cultivars and selections

**Fig 1:**
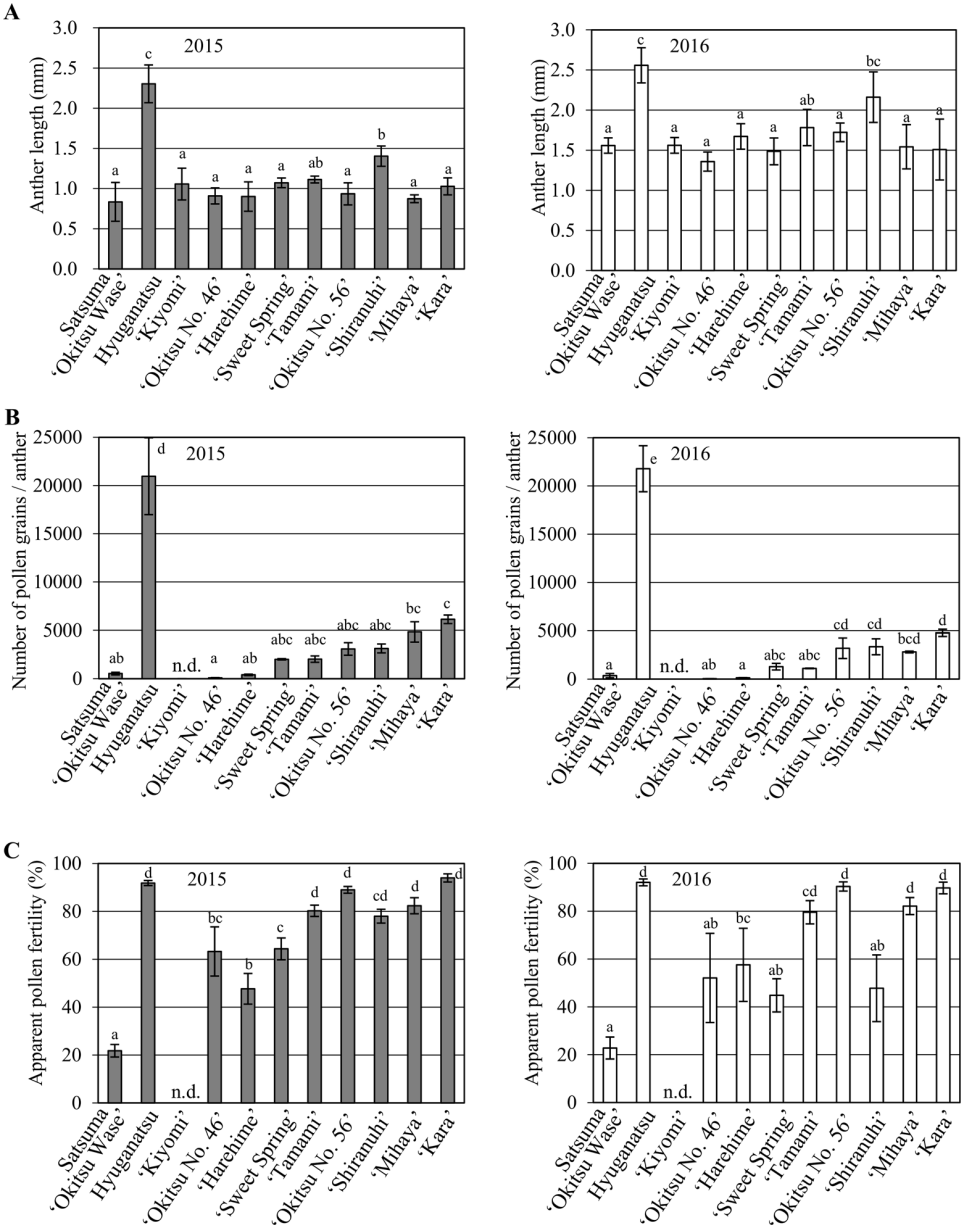
Evaluation of male sterility in cultivars and selections of Satsuma mandarin. (A) Mean anther length (*n* = 5–7); (B) total number of pollen grains per anther; (C) apparent fertility of pollen grains as evaluated for 3–15 anthers per flower by staining with lactophenol blue. Each value represents the average of three biological replicates. Error bars represent the standard deviation. The evaluations were carried out in 2015 and 2016. Bars with the same lower-case letter are not significantly different according to Tukey’s test (*P* < 0.05).

In previous studies, inheritance of male sterility derived from Satsuma has been estimated by evaluating anther development in progeny of Satsuma [9,10]. However, it is widely accepted that the male sterility is caused by either the decreased number of pollen grains in the anther or infertility of the pollen, or both factors. Prior to evaluating male sterility by focusing on the number of pollen grains per anther and pollen fertility, we first determined the size distribution of anther lengths among the citrus cultivars and selections because the size of the anther may affect the absolute number of pollen grains per anther. The anther length was measured in Satsuma ‘Okitsu Wase’ together with ‘Kiyomi’, ‘Okitsu No. 46’, ‘Harehime’, ‘Sweet Spring’, ‘Tamami’, ‘Okitsu No. 56’, ‘Shiranuhi’, ‘Mihaya’ and ‘Kara’, which are derived from Satsuma. Hyuganatsu was used as a control. In 2015, among the Satsuma selected and its progeny, ‘Shiranuhi’ produced the longest anthers, which were about 1.5-fold longer than those of Satsuma ‘Okitsu Wase’ (Fig 1A). The other cultivars derived from Satsuma showed no significant difference in anther length (*P* > 0.05). In contrast, the anthers of hyuganatsu were significantly longer than those of Satsuma and its progeny (about three-fold longer than the anthers of Satsuma ‘Okitsu Wase’) (Fig 1A). The anther length among cultivars and selections showed a similar trend in 2015 and 2016. Though the anther length in 2016 was wholly longer than that in 2015, we considered these differences represented a natural variation due to the sampling date. These observations confirmed that anther length is a relatively stable phenotype within Satsuma and its progeny, and thus is unsuitable for use as an index of male sterility among progeny of Satsuma.

On the basis of these observations, the number of pollen grains per anther and pollen fertility were evaluated in ‘Kiyomi’, ‘Okitsu No. 46’, ‘Harehime’, ‘Sweet Spring’, ‘Tamami’, ‘Okitsu No. 56’, ‘Shiranuhi’, ‘Mihaya’ and ‘Kara’ in 2015 and 2016. In addition, a preliminary evaluation was conducted for rough lemon, sweet orange ‘Trovita’, ‘Meyer lemon’ and hassaku in 2014 and 2016. Both Satsuma ‘Okitsu Wase’ and hyuganatsu were evaluated in 2014, 2015 and 2016, and were treated as a control in these years. No obvious differences in size or color of the pollen grains stained with lactophenol blue were observed among the plant samples (S1 Fig). No pollen grains were detected in ‘Kiyomi’, hence it was classified as ‘no data’ in the statistical analysis (Fig 1B). The number of pollen grains per anther for Satsuma ‘Okitsu Wase’ in 2014, 2015 and 2016 was 279, 529 and 359, respectively, whereas hyuganatsu anthers contained significantly more pollen grains than those of Satsuma (13,755 in 2014, 20,960 in 2015 and 21,779 in 2016; S2A Fig, Fig 1B). These observed values showed similar and comparable trends between the years, which suggested that it is a stable trait. These observations were consistent with general perceptions for these citrus Satsuma is a less pollen, whereas hyuganatsu is a fertility cultivar. Among the progeny of Satsuma examined, ‘Okitsu No. 46’ and ‘Harehime’ showed no significant difference in number of pollen grains per anther compared with that of Satsuma ‘Okitsu Wase’ in 2015 and 2016 (Fig 1B). Although no significant differences were observed in the average number of pollen grains per anther in ‘Sweet Spring’, ‘Tamami’, ‘Okitsu No. 56’, ‘Shiranuhi’ and ‘Mihaya’ compared with that of Satsuma ‘Okitsu Wase’ in 2015, the latter produced fewer pollen grains per anther than the other genotypes. In 2016, no significant differences were observed in the number of pollen grains per anther among ‘Okitsu Wase’, ‘Sweet Spring’ and ‘Tamami’. The number of pollen grains per anther in ‘Okitsu No. 56’, ‘Shiranuhi’ and ‘Mihaya’ was significantly higher than that of ‘Okitsu Wase’. The number of pollen grains per anther in ‘Kara’ was significantly higher than that of ‘Okitsu Wase’ in 2015 and 2016. In addition to the differences observed among Satsuma and its progeny, the number of pollen grains per anther in hyuganatsu was substantially higher than that of all other genotypes examined. When the number of pollen grains per anther was compared among ‘Okitsu No. 46’, ‘Okitsu No. 56’, ‘Kara’ and hyuganatsu, which were used as parents to produce F_1_ populations, ‘Okitsu No. 46’ tended to produce fewer pollen grains per anther than ‘Okitsu No. 56’, although the difference was not significant in 2015. However, the difference was significant in 2016. The number of pollen grains per anther in ‘Kara’ was significantly higher than that of ‘Okitsu No. 46’ in 2015 and 2016. The number of pollen grains per anther in the Satsuma-derived ‘Okitsu No. 46’, ‘Okitsu No. 56’ and ‘Kara’ was markedly less than that in hyuganatsu in 2015 and 2016. Preliminary examination of rough lemon, sweet orange ‘Trovita’, ‘Meyer lemon’, hassaku and Satsuma ‘Okitsu Wase’ in 2014 and 2016 confirmed that the anthers of Satsuma contained significant fewer pollen grains than those cultivars (S2A Fig).

Pollen fertility was expressed as the ratio of densely stained, fully developed pollen grains that were stained dark blue by lactophenol blue, which we previously termed the ‘apparent fertility’ [18]. ‘Kiyomi’ was classified as “no data” because no pollen grains were detected. The apparent pollen fertility of Satsuma ‘Okitsu Wase’ was 21.2% in 2014, 21.8% in 2015 and 22.8% in 2016, and that of hyuganatsu was 86.2% in 2014, 91.8% in 2015 and 92.0% in 2016 (S2B Fig, Fig 1C). The apparent pollen fertility of hyuganatsu was significantly higher than that of Satsuma ‘Okitsu Wase’ in these years. In 2015, the apparent pollen fertility of Satsuma ‘Okitsu Wase’ was significantly lower than the other genotypes. Furthermore, a similar trend of it was observed in 2016 (Fig 1C). The apparent pollen fertility of ‘Okitsu No. 46’, ‘Harehime’ and ‘Sweet Spring’ was 63.2%, 47.7% and 64.4%, respectively, which was significantly higher than that of Satsuma ‘Okitsu Wase’ in 2015. Though a similar trend of it was observed in 2016, no significant differences were observed in it. The apparent pollen fertility of ‘Tamami’, ‘Okitsu No. 56’, ‘Mihaya’ and ‘Kara’ was about 80% or higher in 2015 and 2016. The apparent pollen fertility of ‘Okitsu No. 56’ and ‘Kara’ was approximately 90% and was significantly higher than that of ‘Okitsu No. 46’ in 2015 and 2016. No significant differences were observed among ‘Tamami’, ‘Okitsu No. 56’, ‘Mihaya’, ‘Kara’ and hyuganatsu in 2015 and 2016. In ‘Shiranuhi’, the apparent pollen fertility was 78.0% in 2015 and 47.8% in 2016. In general, the pollen fertility of plants is affected by environmental factor, especially temperature [1]. It is likely that nothing but the pollen fertility of Shiranuhi is susceptible to environmental factor. The comparatively low apparent pollen fertility of Satsuma was confirmed in 2014 and 2016 by comparison of the apparent fertility of Satsuma ‘Okitsu Wase’ with that of rough lemon, sweet orange ‘Trovita’, ‘Meyer lemon’ and hassaku (S2B Fig). These observations indicate that the number of pollen grains and the apparent pollen fertility are stable between years.

Satsuma ‘Okitsu Wase’ showed comparatively few pollen grains per anther and low apparent pollen fertility (Fig 1B and 1C, S2A and S2B Fig). These results were consistent with a previous report that the male sterility of Satsuma is defined by low pollen grain number and low apparent pollen fertility [5]. The number of pollen grains per anther in the progeny of Satsuma was less than half of that observed in rough lemon, sweet orange ‘Trovita’, ‘Meyer lemon’, hassaku or hyuganatsu, which showed little variation in this trait (Fig 1B and S2A Fig). This observation suggests that male sterility among the progeny of Satsuma is primarily caused by the decreased number of pollen grains per anther. Given that all of the Satsuma progeny evaluated in this study share the same organelle genomes derived from Satsuma, these observations strongly suggest that reduced pollen production trait may be regulated by several nuclear genes in Satsuma.

In addition to the dramatic differences in number of pollen grains per anther observed among Satsuma progeny, the apparent pollen fertility of ‘Okitsu No. 46’, ‘Harehime’ and ‘Sweet Spring’ was 50–60%, whereas that of the other genotypes evaluated was similar to hyuganatsu (Fig 1C). This result also raises the possibility that several nuclear genes may contribute to the decrease in apparent pollen fertility. However, the contribution of these genes to apparent pollen fertility is not predicted to account for the decrease in pollen fertility that was observed in Satsuma ‘Okitsu Wase’.

The anther length of hyuganatsu was about twice that of Satsuma and its progeny (Fig 1A). However, the lack of significant differences in anther length observed among Satsuma and its progeny strongly suggests that the differences in pollen grain number was not influenced by anther size. On the basis of these observations, we concluded that the pollen number per anther is not dependent on anther length but is independently regulated by nuclear genes, and would be a good indicator of male sterility of Satsuma.

Prior to evaluation of male sterility in three F_1_ populations (hyuganatsu × ‘Okitsu No. 56’, ‘Okitsu No. 46’ × ‘Okitsu No. 56’ and ‘Okitsu No. 46’ × ‘Kara’), the dispersion among trees in male sterility was evaluated in each Satsuma ‘Okitsu Wase’ and their parents, ‘Okitsu No.46’, ‘Okitsu No. 56’, ‘Kara’ and hyuganatsu (S3A and S3B Fig). The dispersion among trees in the number of pollen grains per anther and the apparent fertility was evaluated statistically with ANOVA. The *p*-value of number of pollen grains per anther in Satsuma ‘Okitsu Wase’, ‘Okitsu No. 46’, ‘Okitsu No. 56’, ‘Kara’ and hyuganatsu was 0.897, 0.664, 0.328, 0.188 and 0.123, respectively. The *p*-value of apparent pollen fertility in Satsuma ‘Okitsu Wase’, ‘Okitsu No. 46’, ‘Okitsu No. 56’, ‘Kara’ and hyuganatsu was 0.217, 0.172, 0.375, 0.605 and 0.152, respectively. This result shows that the number of pollen grains per anther and the apparent pollen fertility are stable trait among trees in each cultivars and selections. Therefore, we investigated only one tree per each individual for the evaluation of male sterility in the F_1_ populations.

### Segregation of male sterility in three F_1_ populations

The mode of inheritance of the reduced pollen production and decreased apparent pollen fertility traits was evaluated with three F_1_ populations (hyuganatsu × ‘Okitsu No. 56’, ‘Okitsu No. 46’ × ‘Okitsu No. 56’ and ‘Okitsu No. 46’ × ‘Kara’). As revealed in the preceding section, ‘Okitsu No. 46’ showed a pollen grain frequency as low as that of Satsuma, and the trait is presumed to be derived from Satsuma. ‘Okitsu No. 56’ also exhibited the reduced pollen production trait, which is next to ‘Okitsu No. 46’ (Fig 1B, S3A Fig).

**Fig 2:**
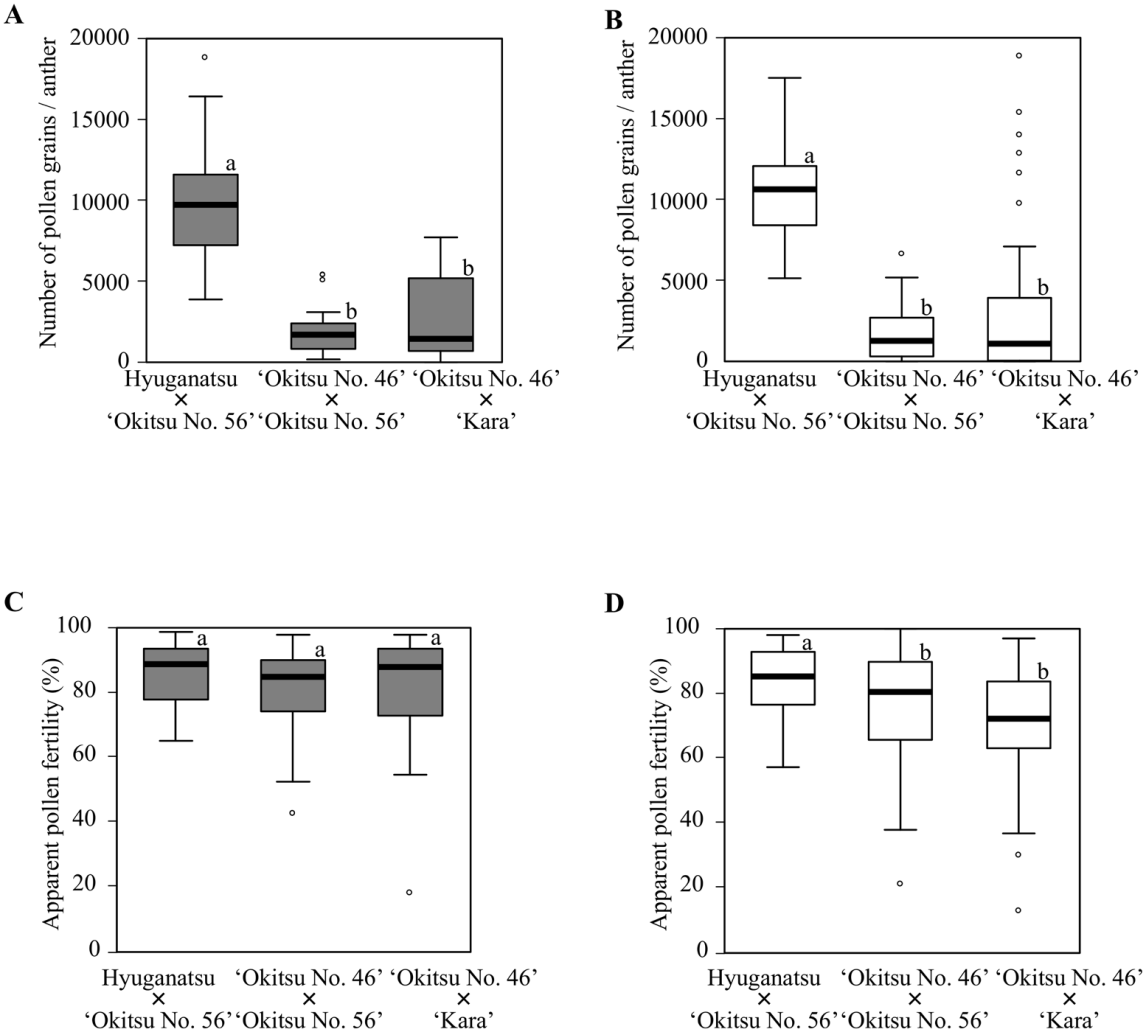
Box plots of pollen grains per anther and apparent pollen fertility of three F_1_ populations. Total number of pollen grains per anther (A) in 2014 and (B) in 2015. Apparent fertility of pollen grains (C) in 2014 and (D) in 2015. The black solid line indicates the median, the box represents the lower and upper quartiles, the upper and lower adjacent lines indicate the minimum and maximum values, and hollow circles represent outliers. Gray boxes represent evaluations in 2014, and white boxes represent data obtained in 2015. Data presented in (A) and (C) were sourced from a previous report [18].

The first flowering of the F_1_ populations was observed in 2014. The flowering individuals were scored for number of pollen grains per anther and apparent pollen fertility individually in 2014 and 2015 (Table 2). Five individuals of the ‘Okitsu No. 46’ × ‘Kara’ population produced no pollen in 2014. Therefore, these five individuals were excluded from the analysis of data obtained in 2014. The number of pollen grains per anther showed segregation within each population, and large differences in the range of the 25th and 75th percentiles were confirmed among these populations. The average number of pollen grains per anther in the hyuganatsu × ‘Okitsu No. 56’ population was significantly greater than that of the ‘Okitsu No. 46’ × ‘Okitsu No. 56’ and ‘Okitsu No. 46’ × ‘Kara’ populations. However, no significant difference was observed between the ‘Okitsu No. 46’ × ‘Okitsu No. 56’ and ‘Okitsu No. 46’ × ‘Kara’ populations in both 2014 and 2015 (Fig 2A and 2B). The apparent pollen fertility of these populations also showed segregation within the population, but no significant differences were observed among the populations in 2014 (Fig 2C). The apparent pollen fertility of the hyuganatsu × ‘Okitsu No. 56’ population was significantly higher than that of the ‘Okitsu No. 46’ × ‘Okitsu No. 56’ and ‘Okitsu No. 46’ × ‘Kara’ populations in 2015 (Fig 2D). However, the median ranged between 72% and 89% among each population (Fig 2D). This result showed that the apparent pollen fertility segregated in the three populations, but the range did not overlap with that of Satsuma.

**Fig 3:**
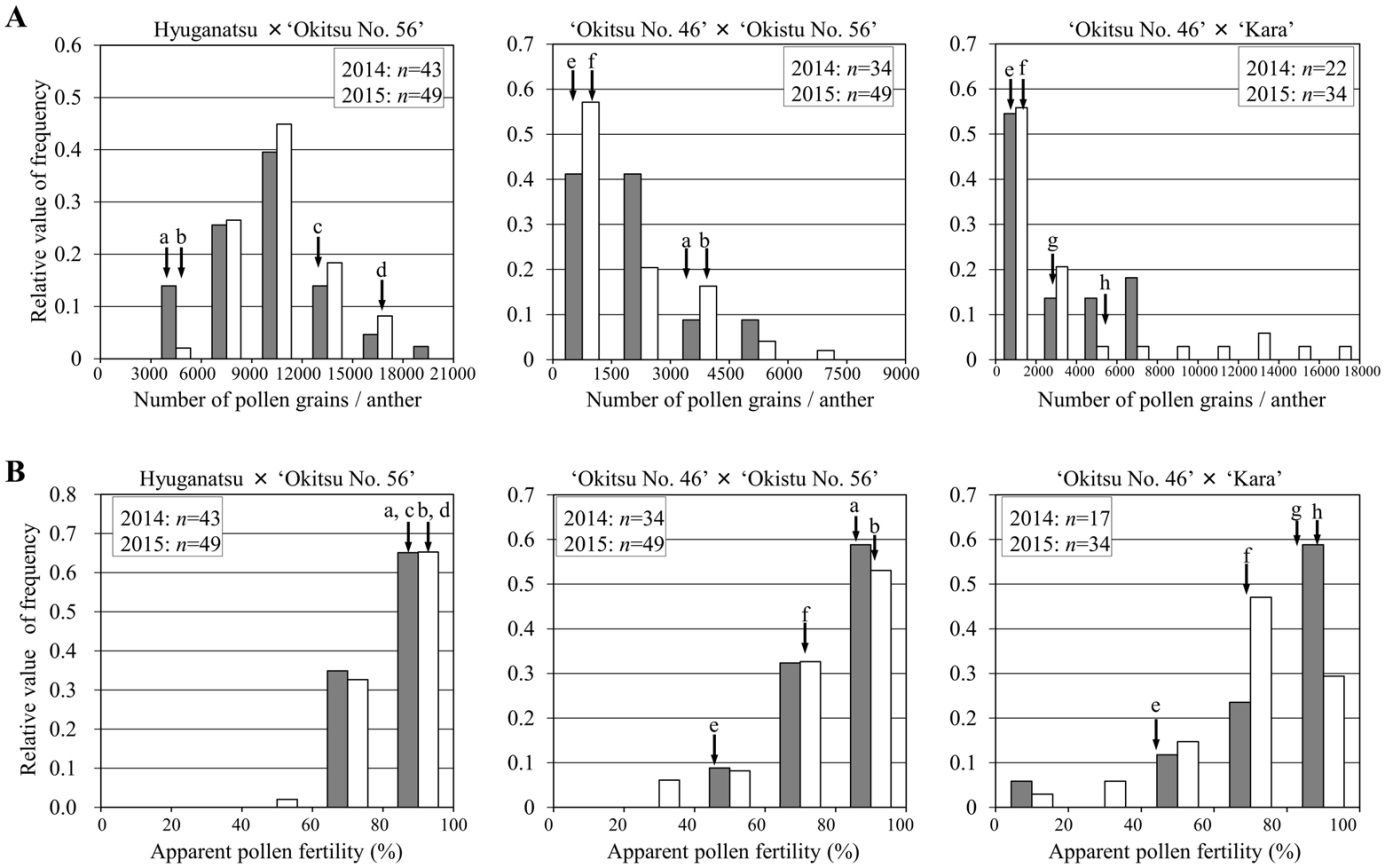
Distribution of pollen grains per anther and apparent pollen fertility of three F_1_ populations. (A) Distribution for number of pollen grains per anther and (B) distribution for apparent pollen fertility. Black arrows indicate the number of pollen grains per anther for each parents in each F_1_ population. a: ‘Okitsu No. 56’ in 2014, b: ‘Okitsu No. 56’ in 2015, c: hyuganatsu in 2014, d: hyuganatsu in 2015, e: ‘Okitsu No. 46’ in 2014, f: ‘Okitsu No. 46’ in 2015, g: ‘Kara’ in 2014, h: ‘Kara’ in 2015. Gray bars represent evaluations in 2014, and white bars represent data obtained in 2015.

With respect to the distribution of the number of pollen grains per anther, the 25th and 75th percentiles showed that the number of pollen grains per anther in the hyuganatsu × ‘Okitsu No. 56’ and ‘Okitsu No. 46’ × ‘Kara’ populations were widely distributed. In contrast, that of the ‘Okitsu No. 46’ × ‘Okitsu No. 56’ population showed a narrower range (Fig 2A and 2B). The frequency distribution of the data for each F_1_ population was also explored by means of histograms (Fig 3). The ‘Okitsu No. 46’ × ‘Okitsu No. 56’ population showed a single peak in number of pollen grains per anther in both 2014 and 2015. The offspring showed segregation, with a considerable number of individuals distributed from that of ‘Okitsu No. 46’ (381 in 2014, 77 in 2015) to that of ‘Okitsu No. 56’ (3,339 in 2014, 3,082 in 2015) (Fig 3A). Half of the progeny (14 individuals in 2014, 28 individuals in 2015) showed a similar number of pollen grains per anther to that of ‘Okitsu No. 46’. In the ‘Okitsu No. 46’ × ‘Kara’ population a single peak was also observed, and the distribution ranged from that of ‘Okitsu No. 46’ (381 in 2014, 77 in 2015) to that of ‘Okitsu No. 56’ (3254 in 2014, 6122 in 2015) (Fig 3A). Half of the progeny (12 individuals in 2014, 19 individuals in 2015) showed a similar number of pollen grains per anther to that of ‘Okitsu No. 46’. Although a wide distribution in number of pollen grains per anther was observed in the hyuganatsu × ‘Okitsu No. 56’ population, the histogram indicated that there is little opportunity to obtain individuals with fewer pollen grains per anther in this population.

The histogram of apparent pollen fertility in the ‘Okitsu No. 46’ × ‘Okitsu No. 56’ population indicated that three and seven individuals showed similar or lower apparent pollen fertility to ‘Okitsu No. 46’ in 2014 and 2015, respectively (Fig 3B). The histogram of apparent pollen fertility in the ‘Okitsu No. 46’ × ‘Kara’ population indicated that three and eight individuals showed similar or lower apparent pollen fertility to ‘Okitsu No. 46’ in 2014 and 2015, respectively (Fig 3B).

The observed number of pollen grains per anther in the ‘Okitsu No. 46’ × ‘Okitsu No. 56’ and ‘Okitsu No. 46’ × ‘Kara’ populations suggests that ‘Okitsu No. 46’ may carry more than one dominant effective gene to decrease the number of pollen grains derived from Satsuma, in contrast to ‘Okitsu No. 56’ and ‘Kara’. The apparent pollen fertility of some individuals in the ‘Okitsu No. 46’ × ‘Okitsu No. 56’ and ‘Okitsu No. 46’ × ‘Kara’ populations was similar to that of ‘Okitsu No. 46’. These results also suggest that ‘Okitsu No. 46’ may possess a gene to suppress pollen development that is derived from Satsuma, and this gene may be inherited by the offspring of ‘Okitsu No. 46’. However, genes that suppress pollen grain development in the progeny may be less effective for development of male-sterile cultivars. Considering the mode of inheritance in the ‘Okitsu No. 46’ × ‘Okitsu No. 56’ and ‘Okitsu No. 46’ × ‘Kara’ populations, it is likely that the number of pollen grains per anther and apparent pollen fertility are quantitative traits. Therefore, it is difficult to discuss exact number of genes which regulate the number of pollen grains per anther and apparent pollen fertility in this study. Accordingly, estimating the genetic effect to decrease the number of pollen grain per anther in detail would contribute to develop a DNA marker to select a male sterile individual.

### Broad-sense heritability of male sterility

The genetic and environmental effects on the number of pollen grains per anther and apparent pollen fertility, and the broad-sense heritability (*h*_*B*_^2^) of both traits were estimated in the three F_1_ populations (S1 and S2 Tables and Table 3). Genetic variance (σ_*g*_^2^) and environmental variance (σ_*e*_^2^) of the number of pollen grains per anther and apparent pollen fertility were estimated by ANOVA from evaluations in 2014 and 2015 (S1 and S2 Tables). The *h*_*B*_^2^ of the number of pollen grains per anther and apparent pollen fertility were estimated from σ_*g*_^2^ and σ_*e*_^2^. The *h*_*B*_^2^ for number of pollen grains per anther was high in the ‘Okitsu No. 46’ × ‘Okitsu No. 56’ and ‘Okitsu No. 46’ × ‘Kara’ populations (Table 3). This result indicates that the number of pollen grains per anther in the ‘Okitsu No. 46’ × ‘Okitsu No. 56’ and ‘Okitsu No. 46’ × ‘Kara’ populations is a genetically regulated stable trait, and is less susceptible to environmental influence. This observation is congruous with our previous report in which we proposed that ‘Okitsu No. 46’ and ‘Okitsu No. 56’ may carry several male sterility genes, and that the genes were inherited by the progeny according to the preliminary evaluation in 2014 [18]. In the present study, however, the *h*_*B*_^2^ for number of pollen grains per anther was markedly lower in the hyuganatsu × ‘Okitsu No. 56’ population compared with that of the other populations. This result suggests that the number of pollen grains per anther in the hyuganatsu × ‘Okitsu No. 56’ population is an unstable trait and that the genetic contribution is restricted. Yamamoto et al. suggest that cytoplasm derived from Satsuma is required to express male sterility [10]. The hyuganatsu × ‘Okitsu No. 56’ progeny carry cytoplasm derived from hyuganatsu, whose cytoplasm differs from that of Satsuma [23]. Given this result and the report of Yamamoto et al. [10], it is likely that in ‘Okitsu No. 56’ Satsuma-type cytoplasm is required for full expression of male sterility. Further evaluation is needed to clarify the relationship between the male sterility gene(s) derived from Satsuma, and the effect of cytoplasm origin in ‘Okitsu No. 56’.

**Table 3:** Broad-sense heritability of number of pollen grains per anther and apparent pollen fertility of three F_1_ populations.

Trait	F_1_ population		
	Hyuganatsu × ‘Okitsu No. 56’	‘Okitsu No. 46’ × ‘Okitsu No. 56’	‘Okitsu No. 46’ × ‘Kara’
Number of pollen grains per anther	0.086	0.898	0.898
Apparent pollen fertility	0.944	0.772	0.895

Broad-sense heritability was calculated as the ratio of genetic variances between genetic and environmental variances (S2 Table).

Considering the frequency distributions and heritability of the number of pollen grains per anther observed in the ‘Okitsu No. 46’ × ‘Okitsu No. 56’ and ‘Okitsu No. 46’ × ‘Kara’ populations, we determined that ‘Okitsu No. 46’ and ‘Okitsu No. 56’ are likely to have inherited nuclear genes for the reduced pollen production trait from Satsuma, and may share some of these genes (Fig 3A and Table 3). The *h*_*B*_^2^ for both ‘Okitsu No. 46’ and ‘Okitsu No. 56’ was 0.898 (Table 3). This value is remarkably high in comparison with previous reports of *h*_*B*_^2^ for fruit trees [20,24–27], although it was estimated from only 2 years of evaluation. Moreover, the mode of inheritance of the reduced pollen production trait in progeny of ‘Okitsu No.46’ indicates its usefulness in citrus breeding to develop novel male-sterile cultivars.

Recent studies focused to the involvement of CMS for the male sterility of Satsuma [12–16,28]. However, the observed segregation and higher heritability for the male sterility in this study strongly suggest the indispensable role of nuclear gene to this important trait. These observations will contribute not only to develop DNA marker linked to the male sterility, but also to clarify the gene-cytoplasmic interaction to cause male sterility in Satsuma. Chae et al reported the development of DNA marker linked to male fertility of *C*. *sunki* [29]. Quantitative trait locus (QTL) analysis of the reduced pollen production trait will clarify the number of genes likely to be involved in regulation of the trait. Developing DNA markers linked to these QTLs in accordance with evaluating the expected contribution of these QTLs will generate DNA markers suitable for marker-assisted selection of male sterility on the basis of decreased number of pollen grains per anther. There are many reports of *h*_*B*_^2^, although the number of field-based studies of plants is much fewer than those on humans and animal [30]. In particular, there are only two reports of *h*_*B*_^2^ of citrus [27,31]. In the present study, we estimated high *h*_*B*_^2^ for the number of pollen grains per anther and apparent pollen fertility. This report will contribute to an improved understanding of the inheritance of male sterility in Satsuma and to estimation of the narrow-sense heritability.

## Conclusion

In this study, we demonstrated that progeny of Satsuma inherited the reduced pollen production trait from Satsuma. This trait is considered to be the primary cause of male sterility in the progeny. Three F_1_ populations (hyuganatsu × ‘Okitsu No. 56’, ‘Okitsu No. 46’ × ‘Okitsu No. 56’ and ‘Okitsu No. 46’ × ‘Kara’) showed strong segregation for number of pollen grains per anther within each population. A considerable number of individuals in the ‘Okitsu No. 46’ × ‘Okitsu No. 56’ and ‘Okitsu No. 46’ × ‘Kara’ populations showed the reduced pollen production trait, which was comparable to that of Satsuma and the parents. The broad-sense heritability for the reduced pollen production trait was remarkably high in these populations. The results suggest that the male sterility in ‘Okitsu No. 46’ is mostly due to the number of pollen grains per anther. This trait is hypothesized to be regulated by more than one dominant nuclear-encoded gene in ‘Okitsu No. 46’. Development of DNA markers linked with male sterility using ‘Okitsu No. 46’ × ‘Okitsu No. 56’ and ‘Okitsu No. 46’ × ‘Kara’ populations is expected to contribute to the breeding of seedless citrus.

## Supporting Information

S1 Fig Pollen grains of cultivars and selections of Satsuma mandarin stained with lactophenol blue. Pollen grains that were stained dark blue were regarded as fully developed, and those that were stained pale blue were regarded as empty pollen grains. Black arrows indicate representative fully developed pollen grains and hollow arrows indicate representative empty pollen grains. Bar = 0.1 mm.
(PDF)

S2 Fig Evaluation of male sterility in citrus cultivars and selections. (A) Total number of pollen grains per anther and (B) apparent fertility of pollen as indicated by lactophenol blue staining. Each value represents the mean of three biological replicates. Error bars represent the standard deviation. The evaluations were carried out in 2014 and 2016. Bars with the same lower-case letter are not significantly different according to Tukey’s test (*P* < 0.05).
(PDF)

S3 Fig The dispersion among trees in male sterility. (A) Total number of pollen grains per anther and (B) apparent fertility of pollen as indicated by lactophenol blue staining obtained from 5 cultivars and selections. Three bars in each cultivars and selections indicate the mean of three biological replicates obtained from individual trees. Error bars represent the standard deviation. The evaluations were carried out in 2016.
(PDF)

S1 Table ANOVA of the number of pollen grains per anther and apparent pollen fertility for 2 years. Data for the number of pollen grains per anther were square-root transformed and data for apparent pollen fertility were arcsine transformed prior to the analysis. In five individuals of the ‘Okitsu No. 46’ × ‘Kara’ population, no pollen grains were detected; therefore, these individuals were excluded from the ANOVA of apparent pollen fertility of the population. σ_*g*_^2^: genetic variance, σ_*y*_^2^: yearly variance, σ_*r*_^2^: residual variance.
(PDF)

S2 Table Estimates of variance components obtained from ANOVA for the number of pollen grains per anther and apparent pollen fertility for 2 years. (PDF)
