# Supplementary figures and images for: Segregation and Heritability of Male Sterility in Populations Derived from Progeny of Satsuma Mandarin
Source: PLoS One. 2016 Sep 2;11(9):e0162408. doi: 10.1371/journal.pone.0162408 (PMC5010215; doi:10.1371/journal.pone.0162408)

**S1 Fig**

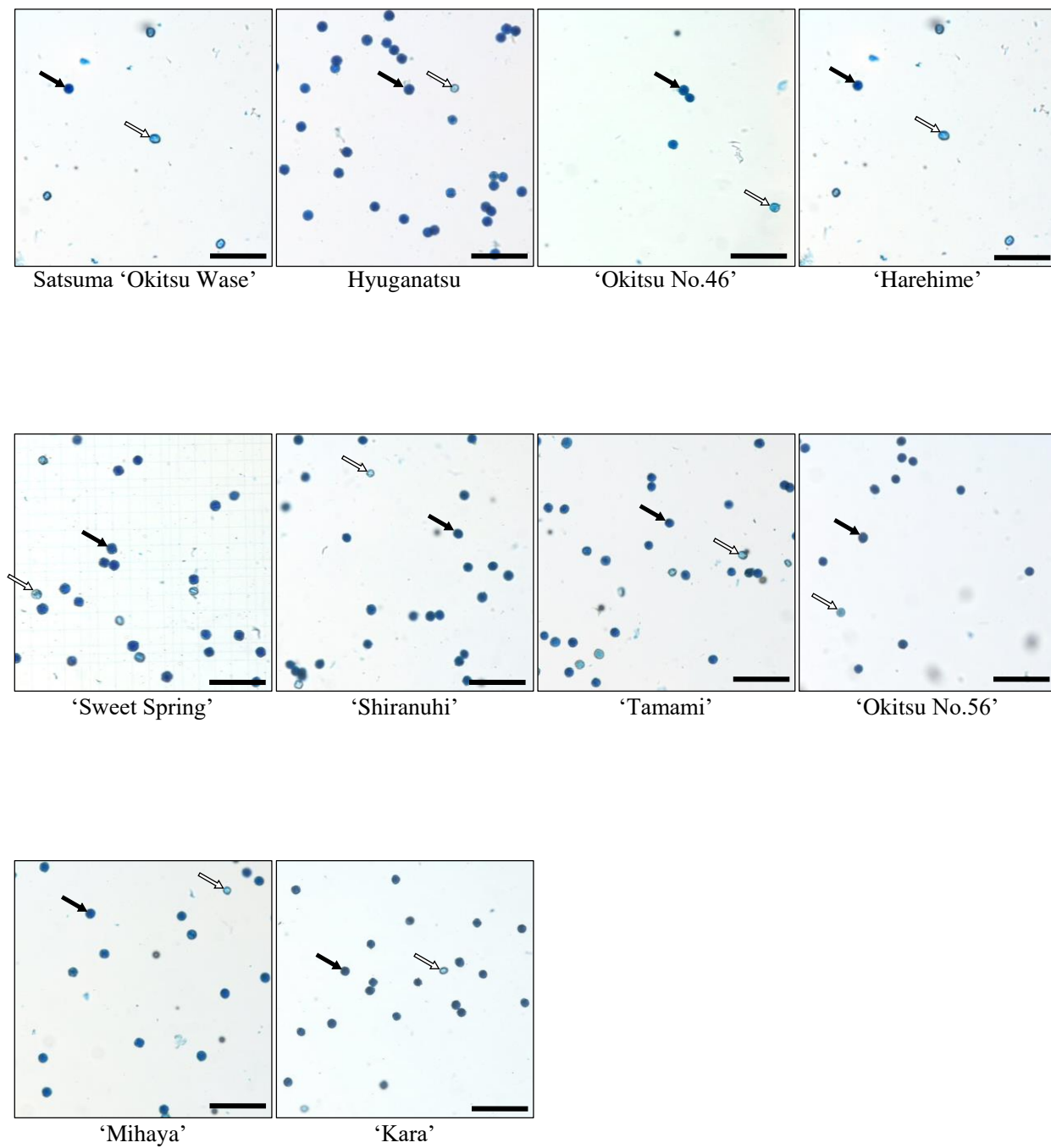

Supplement: S1 Fig — Pollen grains that were stained dark blue were regarded as fully developed, and those that were stained pale blue were regarded as empty pollen grains. Black arrows indicate representative fully developed pollen grains and hollow arrows indicate representative empty pollen grains. Bar = 0.1 mm. (PDF) [file pone.0162408.s001.pdf]

S2 Fig

A

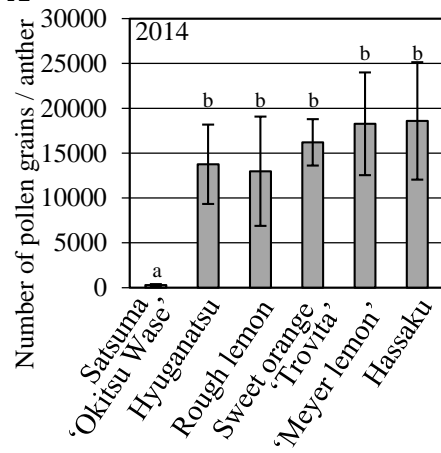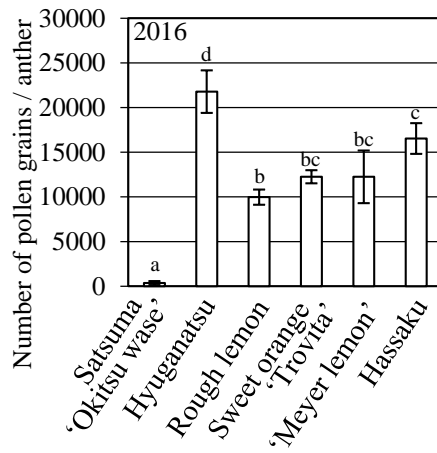

B

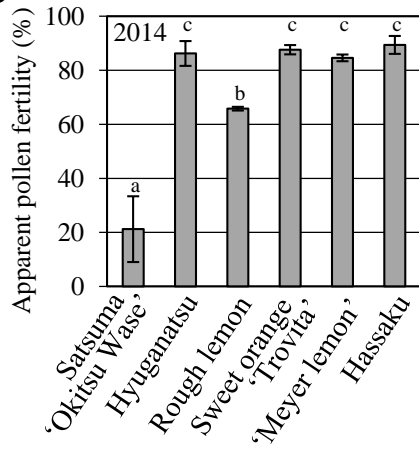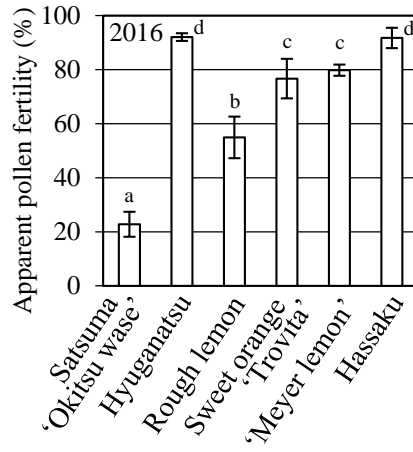

Supplement: S2 Fig — (A) Total number of pollen grains per anther and (B) apparent fertility of pollen as indicated by lactophenol blue staining. Each value represents the mean of three biological replicates. Error bars represent the standard deviation. The evaluations were carried out in 2014 and 2016. Bars with the same lower-case letter are not significantly different according to Tukey’s test (P < 0.05). (PDF) [file pone.0162408.s002.pdf]

S3 Fig

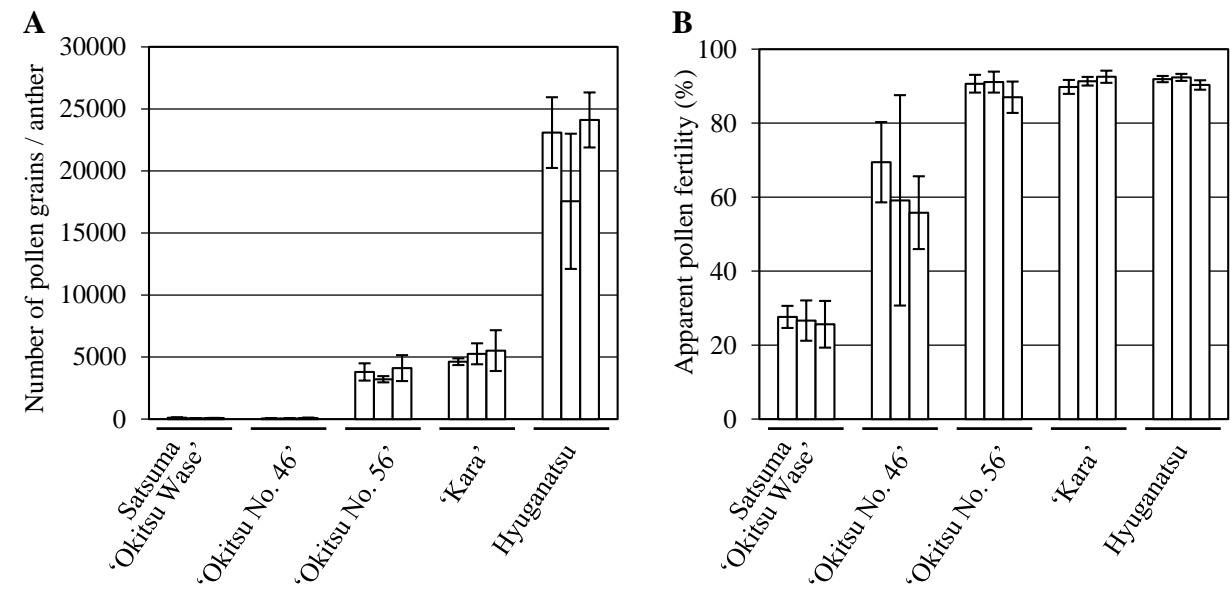

Supplement: S3 Fig — (A) Total number of pollen grains per anther and (B) apparent fertility of pollen as indicated by lactophenol blue staining obtained from 5 cultivars and selections. Three bars in each cultivars and selections indicate the mean of three biological replicates obtained from individual trees. Error bars represent the standard deviation. The evaluations were carried out in 2016. (PDF) [file pone.0162408.s003.pdf]
