# Supplementary material for: Segregation and Heritability of Male Sterility in Populations Derived from Progeny of Satsuma Mandarin
Source: PLoS One. 2016 Sep 2;11(9):e0162408. doi: 10.1371/journal.pone.0162408 (PMC5010215; doi:10.1371/journal.pone.0162408)
Supplement: S1 Table — Data for the number of pollen grains per anther were square-root transformed and data for apparent pollen fertility were arcsine transformed prior to the analysis. In five individuals of the ‘Okitsu No. 46’ × ‘Kara’ population, no pollen grains were detected; therefore, these individuals were excluded from the ANOVA of apparent pollen fertility of the population. σg2: genetic variance, σy2: yearly variance, σr2: residual variance. (PDF) [file pone.0162408.s004.pdf]

S1 Table. ANOVA for the number of pollen grains per anther and apparent pollen fertility evaluated in three F_1 populations in 2014 and 2015.

F_1 population	Trait	Genetic variance	Environmental variance
Hyuganatsu × ‘Okitsu No. 56’	Number of pollen grains per anther	9.1	97.1
	Apparent pollen fertility	109.0	6.5
‘Okitsu No. 46’ × ‘Okitsu No. 56’	Number of pollen grains per anther	296.4	33.7
	Apparent pollen fertility	126.5	37.4
‘Okitsu No. 46’ × ‘Kara’	Number of pollen grains per anther	1283.2	145.9
	Apparent pollen fertility	262.9	31.0
