# Supplementary material for: Segregation and Heritability of Male Sterility in Populations Derived from Progeny of Satsuma Mandarin
Source: PLoS One. 2016 Sep 2;11(9):e0162408. doi: 10.1371/journal.pone.0162408 (PMC5010215; doi:10.1371/journal.pone.0162408)
Supplement: S2 Table — (PDF) [file pone.0162408.s005.pdf]

**S2 Table. Estimates of variance components obtained from ANOVA of the number of pollen grains per anther and apparent pollen fertility evaluated in three $F_1$ populations in 2014 and 2015.**

| $F_1$ population | Trait | Source of variation | df | Sum of squares | Mean square | Expected mean square |
|---|---|---|---|---|---|---|
| Hyuganatsu × 'Okitsu No. 56' | Number of pollen grains per anther | Genotype | 32 | 6445 | 201 | $\sigma_r^2 + 2\sigma_g^2$ |
| | | Year | 1 | 544 | 544 | $\sigma_r^2 + 33\sigma_y^2$ |
| | | Residual | 32 | 5863 | 183 | $\sigma_r^2$ |
| | Apparent pollen fertility | Genotype | 32 | 7389 | 231 | $\sigma_r^2 + 2\sigma_g^2$ |
| | | Year | 1 | 12 | 12 | $\sigma_r^2 + 33\sigma_y^2$ |
| | | Residual | 32 | 416 | 13 | $\sigma_r^2$ |
| 'Okitsu No. 46' × 'Okitsu No. 56' | Number of pollen grains per anther | Genotype | 25 | 16489 | 660 | $\sigma_r^2 + 2\sigma_g^2$ |
| | | Year | 1 | 82 | 82 | $\sigma_r^2 + 26\sigma_y^2$ |
| | | Residual | 25 | 1670 | 67 | $\sigma_r^2$ |
| | Apparent pollen fertility | Genotype | 25 | 8207 | 328 | $\sigma_r^2 + 2\sigma_g^2$ |
| | | Year | 1 | 63 | 63 | $\sigma_r^2 + 26\sigma_y^2$ |
| | | Residual | 25 | 1882 | 75 | $\sigma_r^2$ |
| 'Okitsu No. 46' × 'Kara' | Number of pollen grains per anther | Genotype | 21 | 58427 | 2782 | $\sigma_r^2 + 2\sigma_g^2$ |
| | | Year | 1 | 1887 | 1887 | $\sigma_r^2 + 22\sigma_y^2$ |
| | | Residual | 21 | 4534 | 216 | $\sigma_r^2$ |
| | Apparent pollen fertility | Genotype | 16 | 9363 | 585 | $\sigma_r^2 + 2\sigma_g^2$ |
| | | Year | 1 | 102 | 102 | $\sigma_r^2 + 17\sigma_y^2$ |
| | | Residual | 16 | 951 | 59 | $\sigma_r^2$ |
